# P-36. RSV Vaccination Uptake Among US Adults Aged ≥60 Years Who Are at Increased Risk of Severe RSV Disease (August 2023-February 2024)

**DOI:** 10.1093/ofid/ofae631.243

**Published:** 2025-01-29

**Authors:** Elizabeth M La, Catherine B McGuiness, David Singer, Marie Yasuda, Chi-Chang Chen

**Affiliations:** GSK, Philadelphia, Pennsylvania; IQVIA, Plymouth Meeting, Pennsylvania; GSK, Philadelphia, Pennsylvania; IQVIA, Plymouth Meeting, Pennsylvania; IQVIA, Plymouth Meeting, Pennsylvania

## Abstract

**Background:**

In 2023, two respiratory syncytial virus (RSV) vaccines became available in the United States (US) for adults aged ≥60 years and were recommended by the Centers for Disease Control and Prevention’s Advisory Committee on Immunization Practices using shared clinical decision-making (i.e., requiring joint decision-making between healthcare professionals and their patients). This study evaluated RSV vaccination uptake through February 2024, overall and by specific risk factors for severe RSV disease.

**Figure 1.**
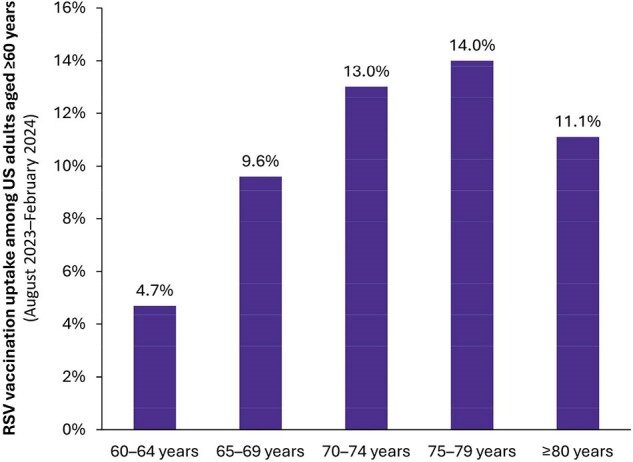
Age-specific RSV vaccination uptake among US adults aged ≥60 years RSV = respiratory syncytial virus; US = United States.

Note: Patients with data quality issues or who received an RSV vaccine before August 1, 2023 were excluded from analyses.

**Methods:**

A retrospective database analysis evaluated RSV vaccination uptake between August 1, 2023-February 29, 2024, using IQVIA’s open-source pharmacy (LRx) and medical (Dx) claims data. The study included patients aged ≥60 years who had ≥1 claim between January 1, 2023-February 29, 2024. Monthly and cumulative uptake were assessed as the number and percentage of eligible older adults who received an RSV vaccine during the study period, with cumulative uptake stratified by potential risk factors for severe RSV disease (e.g., chronic pulmonary or cardiovascular conditions). Descriptive analyses were based on observed vaccination claims (data were not projected to US population).

**Figure 2.**
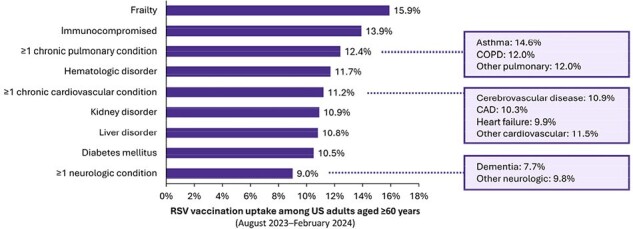
RSV vaccination uptake among US adults aged ≥60 years, by potential risk factors for severe RSV disease CAD = coronary artery disease; CCI = Charlson Comorbidity Index; COPD = chronic obstructive pulmonary disease; RSV = respiratory syncytial virus; US = United States.

Note: Patients with data quality issues or who received an RSV vaccine before August 1, 2023 were excluded from analyses. Potential risk factors for severe RSV disease were identified based on the presence of relevant diagnosis and/or procedure codes in claims prior to August 1, 2023 (i.e., using all available history). For the analysis by frailty status, patients were required to have claim activity (both medical [Dx] and pharmacy [LRx]) and stability during the 1-year period prior to August 1, 2023. Frailty status was evaluated based on the presence of a diagnosis for age-related physical debility and/or cumulative frailty score ≥2 based on age and CCI score (using all available history). For age, patients received a score of 0 if aged ≤75 years, 1 if aged 76-80 years, and 2 if aged ≥81 years. For CCI, patients received a score of 0 if CCI ≤1 and 1 if CCI ≥2.

**Results:**

Nearly 9.2 million older adults (10.0%) received an RSV vaccination between August 2023-February 2024, with 26.1% of vaccinations occurring in October. Cumulative uptake by age was lowest among adults aged 60-64 years (4.7%) and highest among adults aged 75-79 years (14.0%) (Figure 1). RSV vaccination uptake was higher among older adults with ≥1 potential risk factor for severe RSV disease (11.5%) versus those without any risk factors (7.9%). Among evaluated risk factors, uptake ranged from a low of 7.7% for older adults with dementia to a high of 15.9% for frail older adults (Figure 2).

**Conclusion:**

Approximately 1 in 10 adults aged ≥60 years received an RSV vaccination during the first season of vaccine availability, with relatively low uptake observed across ages and evaluated risk factors. Previous research has estimated that nearly half of older adults have a diagnosed risk factor for severe RSV disease, suggesting that additional efforts are needed to support RSV prevention among older adults at highest risk.

**FUNDING:** GSK (GSK study identifier: VEO-000828)

**Disclosures:**

**Elizabeth M. La, PhD**, GSK: employee|GSK: Stocks/Bonds (Private Company) **Catherine B. McGuiness, MA, MS**, GSK: Advisor/Consultant **David Singer, PharmD, MS**, GSK: employee|GSK: Stocks/Bonds (Public Company) **Marie Yasuda, PharmD, MS**, Amgen: Grant/Research Support|Bayer: Grant/Research Support|BMS: Grant/Research Support|GSK: Advisor/Consultant|Novartis: Grant/Research Support|Sandoz: Grant/Research Support|Servier: Grant/Research Support **Chi-Chang Chen, PhD, MSPharm**, Amgen: Grant/Research Support|Bayer: Grant/Research Support|BMS: Grant/Research Support|GSK: Advisor/Consultant|Novartis: Grant/Research Support|Regeneron: Grant/Research Support

